# Recombinant hNeuritin Promotes Structural and Functional Recovery of Sciatic Nerve Injury in Rats

**DOI:** 10.3389/fnins.2016.00589

**Published:** 2016-12-22

**Authors:** Haiyan Wang, Xinli Li, Liya Shan, Jingling Zhu, Rong Chen, Yuan Li, Wumei Yuan, Lei Yang, Jin Huang

**Affiliations:** ^1^The Key Laboratory of Xinjiang Endemic and Ethnic Diseases and Department of Biochemistry, Shihezi University School of MedicineShihezi, China; ^2^Laboratory Medicine Department of Sixth People's Hospital of ChengduChengdu, China; ^3^Occupational and Environmental Health, Department of Preventive Medicine, School of Medicine, Hangzhou Normal UniversityHangzhou, China; ^4^Department of Otolaryngology Head and Neck Surgery, The Affiliated Hospital of Hangzhou Normal UniversityHangzhou, China

**Keywords:** neuritin, sciatic nerve injury, restoration, walking-track test, growth-associated protein 43, neurofilament 200

## Abstract

Neuritin is a new neurotropic factor implicated in nervous system development and plasticity. Studies have shown that Neuritin is upregulated in injured nerves, suggesting that it is involved in nerve repair. To test this hypothesis, we investigated whether recombinant human Neuritin could restore nerve structure and function in a rat model of sciatic nerve injury. Neuritin treatment had a dose-dependent effect on functional recovery 4 weeks after injury, as determined by the walking-track test. Similar trends were observed for gastrocnemius muscular strength and nerve conduction velocity. Additionally, sciatic nerve fiber density and organization as well as degree of remyelination were increased, while growth-associated protein 43 and neurofilament 200 expression was upregulated upon treatment with Neuritin. These findings demonstrate that Neuritin stimulates nerve regeneration and functional recovery and thus promotes the repair of injured sciatic nerves.

## Introduction

Neurotrophic factors supported the migration, growth, and survival of neurons. These secreted factors are also critical for the maintenance and plasticity of the adult nervous system and are of clinical interest since they can serve as therapeutic agents in the treatment of neurodegenerative disorders and nerve injury (Vilar and Mira, 2016). Neuritin is a neurotropic factor that has been implicated in nervous system development and plasticity (Nedivi et al., 1993). It is highly expressed in the developing nervous system, with the level decreasing upon maturation (Naeve et al., 1997). However, Neuritin is upregulated in adult neural structures, such as the olfactory bulb, hippocampus, visual cortex, and Purkinje fibers (Nedivi et al., 1996, 2001; Yamagata et al., 1998) as well as during transient global ischemia, traumatic brain injury, spinal cord injury, and androgen treatment (Di Giovanni et al., 2005b; Han et al., 2007; Fargo et al., 2008; He et al., 2013). The latter was shown to promote functional recovery of injured facial nerves, which was associated with Neuritin upregulation; conversely, blocking androgen reduced Neuritin expression, suggesting a correlation between Neuritin, and functional recovery of injured facial nerves (Fargo et al., 2008).

Neuritin is a secreted monomeric protein that promotes neurite outgrowth and the stabilization and maturation of synapses in cultured neurons (Nedivi et al., 1998; Cantallops et al., 2000; Javaherian and Cline, 2005). Neuritin overexpression induces the recovery of crushed optic nerve in mouse (Sharma et al., 2015). We recently showed that recombinant Neuritin protects neurons against apoptosis following spinal cord injury (Gao et al., 2016). In the present study, we investigated whether Neuritin can restore nerve structure and function in a rat model of sciatic nerve injury. The results reveal that recombinant hNeuritin accelerates the functional recovery and regeneration of injured sciatic nerves, highlighting its potential therapeutic application.

## Materials and methods

### Ethics statement

Animal experiments were carried out in accordance with the National Institute of Health Guide for the Care and Use of Laboratory Animals. The study protocol was approved by the Animal Subjects Review Board of the First Affiliated Hospital of Shihezi University School of Medicine (permit no. 2008LL11). Surgeries were carried out under sodium pentobarbital anesthesia.

### Generation of recombinant hNeuritin protein

*Pichia pastoris* strain GS115 (Invitrogen) and the P. *pastoris* expression vector pPIC9K (Invitrogen, Carlsbad, CA, USA) were used to produce recombinant hNeuritin protein. The hNeuritin fragment (without the glycosylphosphatidylinositol anchor) was cloned into pPIC9K with a 6 × His tag at the 5′-end. The GS115/pPIC9K-His-Neuritin plasmid was stored at −80°C until use (Zhang et al., 2015). For protein expression, cells harboring GS115/pPIC9K-His-Neuritin were thawed in yeast extract peptone dextrose medium (220 rpm, 30°C, 10–16 h), then transferred to BMGY medium [1%(v/v)] and cultured under the same conditions until the optical density exceeded 2.0. The cell suspension was centrifuged (6000 rpm, 5 min), and the precipitates was collected and dissolved in BMMY medium containing 1% methanol to induce His-Neuritin expression (220 rpm, 30°C, 72 h); 100% methanol was added for 24 h to a final concentration of 1%. Then the supernatant containing His-Neuritin was collected by centrifugation (14,000 rpm, 4°C, 30 min), then mixed to 65% saturation with (NH_4_)_2_SO_4_ while stirring (4 °C, 12 h). The following day, the protein sample was collected by centrifugation (14,000 rpm, 4°C, 30 min) and the precipitate was dissolved in the chromatography solution, which was applied to a HisTrap FF Crude pre-packed column (GE Healthcare Life Sciences, Little Chalfont, UK). Eluted protein samples were dialyzed to reduce the salt concentration and concentrated using Amicon Ultra-15 filter units (Millipore, Billerica, MA, USA). Protein concentrations were determined by the Bradford method (Bio-Rad Protein Assay; Bio-Rad, Hercules, CA, USA), and expression was confirmed by sodium dodecyl sulfate polyacrylamide gel electrophoresis (SDS-PAGE) and Western blot using a mouse anti-His antibody (1:500, CW 0257A; cwbiotech, Shanghai, China). Protein purity was determined by SDS-PAGE as the optical density of the Neuritin band divided by that of total protein in the same lane. Protein solutions were lyophilized at −56°C in a freeze dryer for subsequent assays (FD5510; SIM International, USA).

### Treatment of PC12 cells with recombinant hNeuritin

PC12 cells were cultured in Roswell Park Memorial Institute 1640 medium, 5% fetal bovine serum, and 10% horse serum (Gibco, Grand Island, NY, USA) at 10^6^/35 mm dish as previously described (Green, 1977). The PC12 cells were passaged and seeded onto poly-L-lysine-coated dishes at a density of 10^5^/35 mm dish. Known volumes of medium containing hosphate-buffered saline (PBS; negative control), 6 × His protein (to exclude the effect of His tag, 1 μg/ml, Bioss, China), and the purified recombinant hNeuritin protein (1 μg/ml) were added; the PBS control group was treated with an equal volume of His protein. Morphological changes in the cells were observed 3 days after treatment under an inverted phase-contrast microscope (Olympus, Tokyo, Japan), and five random fields were imaged per dish; samples for each group were prepared in 3 replicates and the experiment was repeated three times. Neurites were analyzed using ImageJ software (National Institutes of Health, Bethesda, MD, USA). To exclude variability in the number of cells per field, neurites were quantified as neurite length/cell.

### Animals

Wistar rats of both sexes (*n* = 80; 12-week-old) weighing 200 g to 220 g were obtained from the Institute of Epidemiology, Xinjiang Uygur Autonomous Region under standardized laboratory conditions in an air-conditioned room at constant temperature (23°C ± 2°C) on a 12:12-h light/dark cycle with free access to food and water. The animals were randomly assigned to one of the following four groups (*n* = 20 each): sham operation, normal saline (NS) group, 3-μg Neuritin (N3), and 5-μg Neuritin (N5). Five rats were selected for testing at each time point.

### Sciatic nerve injury model

Rats were fixed in a prone position and anesthetized by intraperitoneal injection of 0.5% sodium pentobarbital (40 mg/kg body weight, Sigma-Aldrich, St. Louis, CA, USA). A 3-cm longitudinal incision was made under sterile conditions in the posterolateral area of the left buttock and the left sciatic nerve was fully exposed by blunt dissection. In the sham group, surgery stopped at the fascia before the skin was closed. The remaining groups were subjected to severe clamp injury by application of pressure onto sciatic nerve with gearless mosquito forceps (one ratchet of the forceps) at a point 0.5 cm below the piriformis for 30 s. A damaged section 5 mm long was observed after clamping in which the nerve fibers were ruptured but the epineurium was left intact. The nerve tissue became thin at the lesion site, which was defined as neurotmesis according to the Seddon classification. We marked the epineuriums with 9–0 microsutures, reset the nerves, applied gelatin sponges (0.5 × 0.5 × 0.5 cm) infiltrated with NS or different concentrations of Neuritin to the site of injury, and separately sutured the fascia and skin (Smith et al., 1993). After the operation the rats showed immediate behavioral signs of nerve injury; that is, the ipsilateral leg and foot muscles were completely paralyzed; the claw reflex disappeared; and the lower extremities showed claudication, with toes that were completely closed.

### Neuritin treatment

The groups were treated as follows: sham rats underwent sham operation only; rats in the NS group were subjected to sciatic nerve injury and gelatin sponges that were infiltrated with 1 ml NS; and rats in the N3 and N5 groups were subjected to sciatic nerve injury and gelatin sponges that were infiltrated with Neuritin at 1.5 and 2.5 μg/100 g body weight, respectively, in 1 ml NS (for doses of 3 and 5 μg, respectively). These doses of Neuritin did not cause cytotoxity (Li et al., 2008). All animals except those in the sham groups received daily intramuscular injections of Neuritin at the injury site for 1 week after the operation at the indicated doses. The frequency of injection was then changed to every other day for 3 weeks and then to every 3 days for 2 weeks.

### Assessment of sciatic nerve function

Walking tests were performed 2, 4, 6, and 8 weeks after sciatic nerve injury to determine the sciatic nerve function index (SFI). After dipping the hind feet in carbon ink, each rat was placed at one end of a paper-lined walkway; four or five footprints were left on the paper when rat walked to the other end. Sufficiently clear footprints from experimental (prefix E) and normal (prefix N) limbs were evaluated according to three parameters: foot print length (PL; distance from the heel to the third toe); toe spread (TS; distance from the first to fifth toe); intermediary toe spread (IT; distance from the second to the fourth toe). The Bain–Mackinnon–Hunter SFI was then calculated using the formula (Hare et al., 1992):

$$\begin{array}{l} \text{SFI}=-38.3\;\left[\left(\text{EPL}-\text{NPL}\right)/\text{NPL}\right]+109.5\;\left[\left(\text{ETS}-\text{NTS}\right)/\text{NTS}\right]\\ +13.3\;\left[\left(\text{EIT}-\text{NIT}\right)/\text{NIT}\right]-8.8 \end{array}$$

The test was repeated three times and the average of three readings was analyzed. SFI values ranged from −100 to 0, with 0 indicating normal or complete recovery of nerve function and −100 indicating total loss of function (Bain et al., 1989).

### Electrophysiology

Electrophysiological recordings were carried out 2, 4, 6, and 8 weeks after sciatic nerve injury. After the walking tests, five rats in each group were anesthetized by intraperitoneal injection of 3% sodium amobarbital (40 mg/kg body weight, Sigma-Aldrich). A longitudinal incision was made under sterile conditions in the posterolateral part of both legs; the skin and muscles were separated layer by layer to fully expose the sciatic nerve, which were kept wet during recording that were carried out at room temperature (~25°C) using a BL-420E multifunctional biological signal recording system (Chengdu TME Technology, Chengdu, China). Sciatic nerves from the injured and contralateral uninjured sides were exposed and a ground electrode was connected to metal clamps was attached to the cut edge of the tissue. Stimulating and recording electrodes were hooked proximal and distal to the injury site, respectively, with good contact with the nerve stem. Nerve conduction velocity (NCV) was assessed by inducing a compound action potential from the stimulating electrode at the proximal end and recording from the recording electrode at the distal end of the injury site. NCV was calculated as the impulse conduction distance divided by conduction time. The degree of recovery of NCV (experimental data/control data × 100%) was calculated using the contralateral side as an internal control. Experimental and control data were obtained from the lesion and contralateral sides, respectively.

### Evaluation of gastrocnemius muscular strength

At 2, 4, 6, and 8 weeks after the electrophysiological testing, the gastrocnemius muscle in both legs were cut at the end of the Achilles tendons, and the muscles (1 cm length) were separated and connected to a strength transducer via a 1–0 silk suture. The maximal tetanized contractile strength of each sciatic nerve was recorded at a maximum stimulus intensity of 50 Hz and pulse width of 2 ms for 3 s. The degree of recovery of gastrocnemius muscular strength was calculated as (experimental data/control data) × 100%.

### Morphological analysis

At 2, 4, 6 and 8 weeks after injury, five rats from each group were sacrificed by cervical dislocation and the entire sciatic nerve from both injured and uninjured sides were collected and fixed in a 10% paraformaldehyde solution for 24 h. Segments 2 mm in length were removed from an area 2 and 4 mm distal to the lesion site. The tissues were embedded in paraffin and 7-μm serial sections were cut for hematoxylin and eosin (H&E) staining, Sevier-Munger silver staining (AgNO_3_, Shenbo Chemical, Shanghai, China), and myelin staining with Solechrome Cyanin R staining (Like Trading, Guangzhou, China). Image-Pro Plus software (Media Cybernetic, Rockville, MD, USA) was used to obtain average counts of myelinated fibers (μm^−2^) in five randomly selected fields per group.

### Immunohistochemical analysis

Paraffin-embedded sciatic nerve tissue specimens were cut at a thickness of 5 μm and the sections were examined for expression of growth associated protein (GAP) 43 and neurofilament (NF) 200. Sections were rinsed three times with water and incubated in 0.3% hydrogen peroxide for 10 min, in 0.01 M citric acid for 10 min, and goat serum for 20 min. The sections were then incubated overnight with monoclonal antibodies against GAP43 (1:1200, ab50608) or NF200 (1:1000, ab24574) followed by incubation with a goat anti-mouse IgG (Bio-Rad), and then visualized by treatment with a biotin-avidin-peroxidase complex using diaminobenzidine tetrahydrochloride as the chromogen (Bio-Rad, China). Sections were counterstained with hematoxylin for 2 min.

### Statistical analysis

Results are expressed as mean ± standard deviation. Differences between two groups (NS vs. N5, or NS vs. N3) and among groups were evaluated with the least significant difference (LSD) *post-hoc* test and analysis of variance, respectively. *P* < 0.05 was considered statistically significant.

## Results

### Identification of recombinant hNeuritin protein

**Figure 1:**
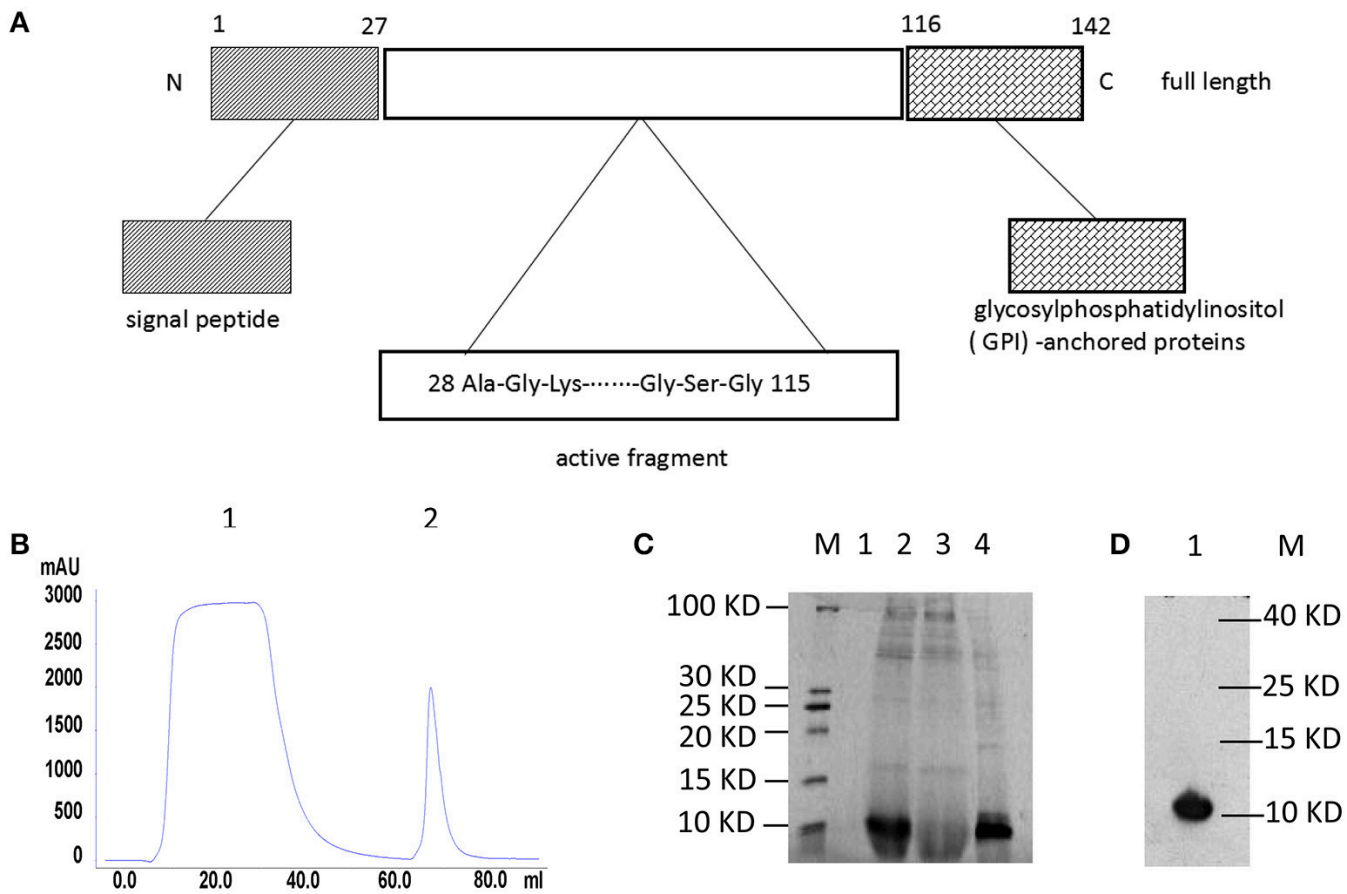
**Structure and purification of recombinant hNeuritin. (A)** Schematic illustration of hNeuritin protein structure. Neuritin is a small glycoprotein of 142 amino acids; the soluble form used in this study is generated via cleavage of the signal peptide. **(B)** Affinity chromatography elution profile of the hNeuritin. Peaks 1 represents effluent and peak 2 is the eluted protein. **(C)** SDS-PAGE analysis of recombinant hNeuritin. M, protein marker; lane 1, culture supernatant before methanol induction; lane 2, culture supernatant after 3 days of methanol induction; lane 3, protein effluent; lane 4, eluted protein. **(D)** Western blot analysis of recombinant hNeuritin. M, protein marker; lane 1, protein eluted with anti-His antibody (~11 kDa).

The structure of **hNeuritin** protein is shown in Figure 1A. Soluble recombinant hNeuritin was collected by linear elution by monitoring the major protein absorption peaks when flows through a HisTrap column packed with Ni Sepharose 6 Fast Flow Crude (Figure 1B). SDS-PAGE analysis indicated that the protein was highly expressed, with a purity >95% (Figure 1C). Western blot analysis using an anti-His monoclonal antibody confirmed the identity of the protein as recombinant hNeuritin, which had a molecular weight of ~11 kDa (Figure 1D), as confirmed by Coomassie staining.

### Neuritin promotes neurite outgrowth in PC12 cells

**Figure 2:**
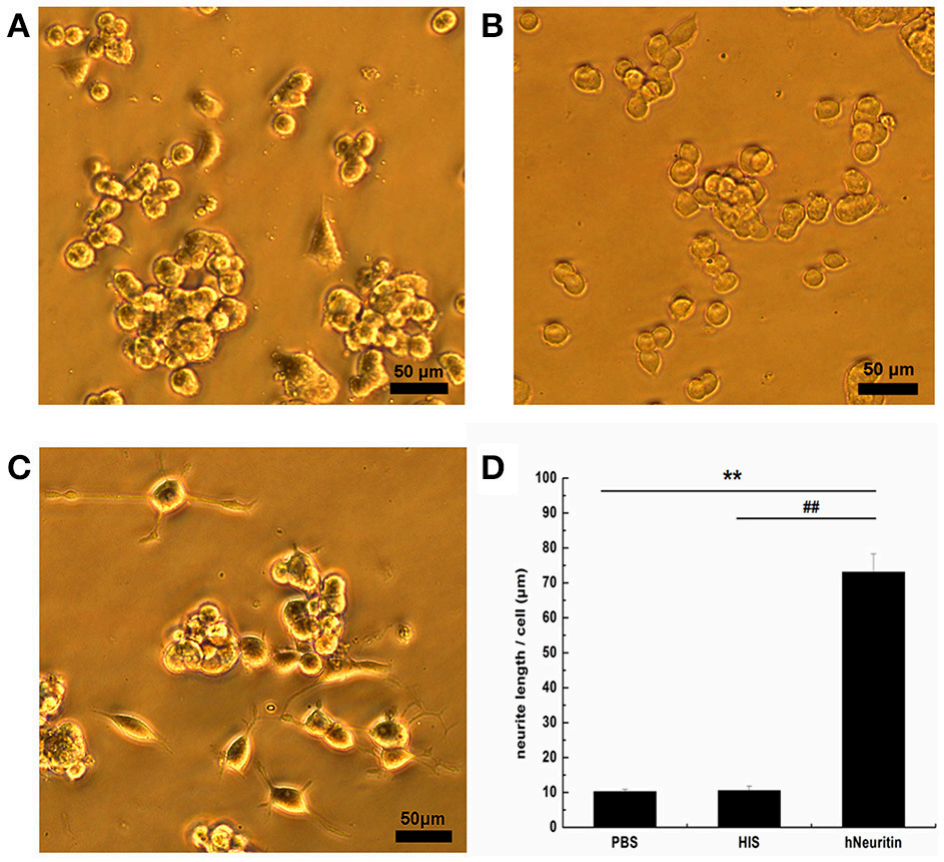
**Effects of Neuritin on PC12 cells. (A–C)** Cell morphology in the PBS control **(A)**, 6 × His protein control **(B)**, and recombinant hNeuritin **(C)** after 3 days of incubation. **(D)** Quantification of neurite length (*n* = 3). ^**^P < 0.01 vs. PBS group; ^*##*^*P* < 0.01 vs. 6 × His protein group. Scale bar = 50 μm.

PBS, 6 × His protein, and purified Neuritin were added to the medium of PC12 cells for 3 days. Cell morphology was observed under an inverted phase contrast microscope. Small, round PC12 cells lacking neurites were observed in the PBS (Figure 2A) and 6 × His (Figure 2B) control groups. In contrast, PC12 cells treated with recombinant hNeuritin showed a network of neurites (Figure 2C), demonstrating that the protein was biologically active (*P* < 0.01). Increase in neurite formation in the presence of Neuritin was confirmed by quantitative analysis of neurite number (Figure 2D).

### Neuritin accelerates functional recovery of injured sciatic nerve

**Figure 3:**
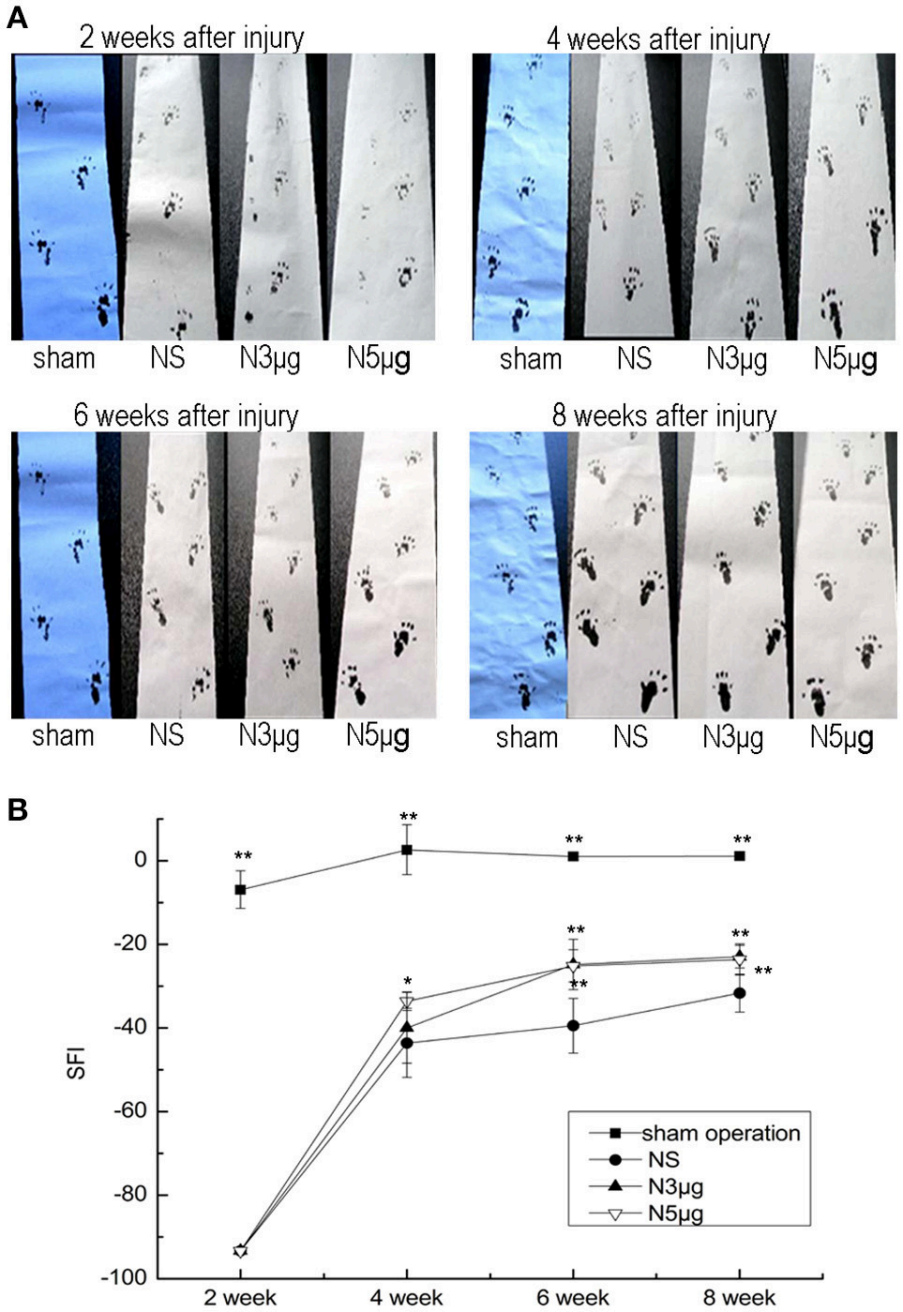
**Functional recovery after sciatic nerve injury. (A)** Walking-track analysis of rats at weeks 2, 4, 6, and 8 after sciatic nerve injury. **(B)** SFI showing the recovery of injured sciatic nerve at indicated time points (*n* = 5). ^*^P < 0.05, ^**^P < 0.01 vs. NS group.

A walking-track test was carried out to determine the effects of Neuritin on the recovery of sciatic nerve function (Figure 3A). SFI was lower in all experimental groups as compared to the sham group at each time points (*P* < 0.01), indicating limb dysfunction following nerve injury (Figure 3B). Nerve function began to recover at 4 weeks, with a significant difference appearing between the N5 and NS groups (N5: −33.61 ± 2.24, NS: −43.59 ± 8.30; *P* < 0.05). Although N5 and N3 groups showed similar trends in recovery of SFI values, these were similar between N3 (−39.98 ± 8.49) and NS groups. At week 6, nerve function was higher in both Neuritin-treated groups than in the NS group (N5: −25.16 ± 3.83, N3: −24.80 ± 6.02, NS: −39.49 ± 6.53; *P* < 0.01). At week 8, the SFI values were higher in all experimental groups, but a significant difference was observed between the Neuritin and NS groups (*P* < 0.01). These results indicate that Neuritin treatment accelerates functional recovery of rat sciatic nerve after injury.

### Assessment of gastrocnemius muscular strength

**Figure 4:**
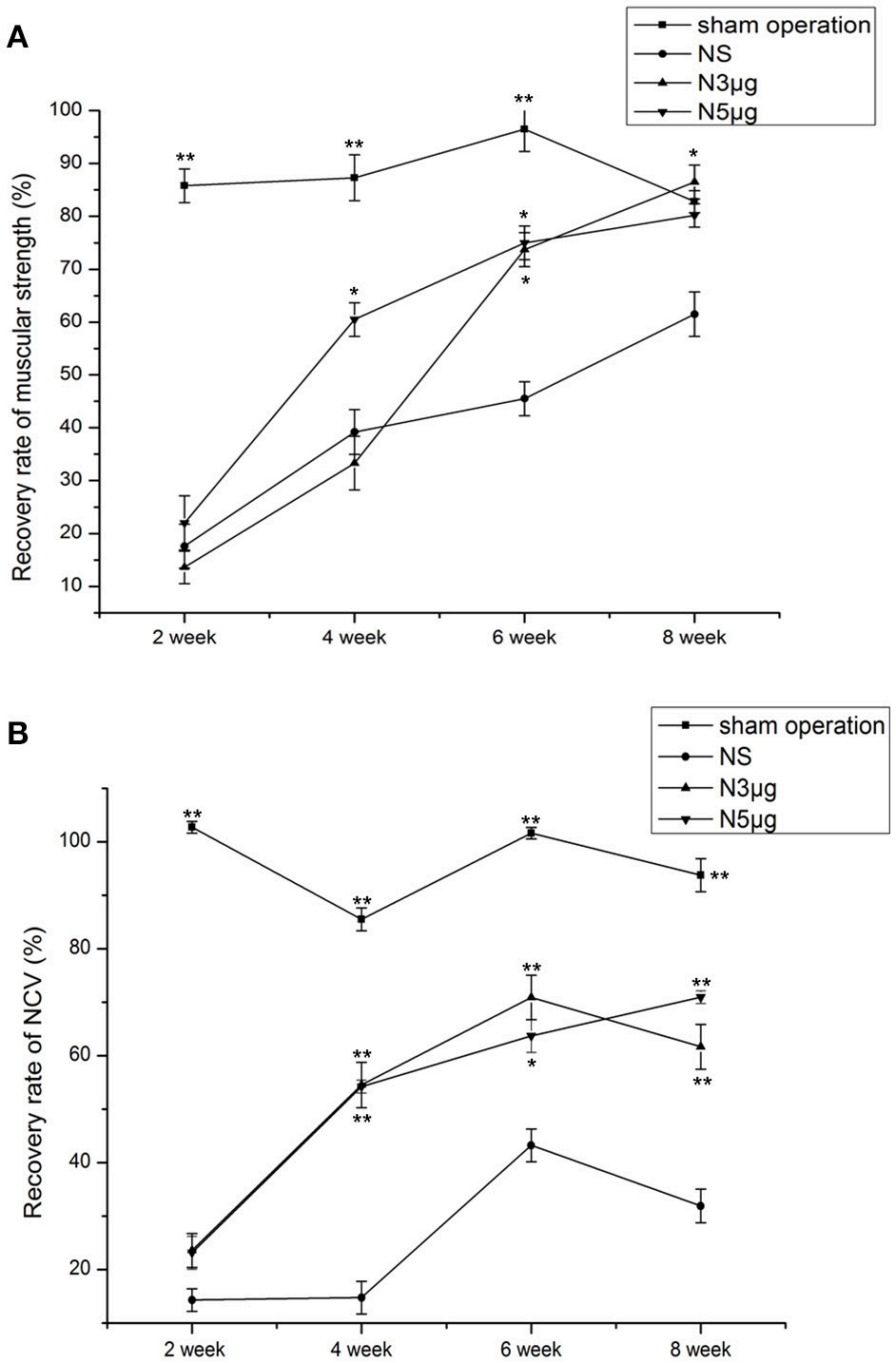
**Functional recovery in the sciatic nerve at different time points after injury, as determined by electrophysiological recordings. (A)** Recovery of gastrocnemius muscular strength at weeks 2, 4, 6, and 8 after nerve injury. **(B)** Recovery of NCV at weeks 2, 4, 6, and 8 after nerve injury. The degree of recovery of gastrocnemius muscular strength and NCV were calculated as experimental data/control data × 100%. ^*^P < 0.05, ^**^P < 0.01 vs. NS group.

Gastrocnemius muscular strength was reduced in all experimental groups relative to the sham group from week 2 to week 6 (*P* < 0.01), consistent with loss of muscle innervation (Figure 4A). However, muscular strength recovered by the 4-week time point, with a significant difference between the N5 and NS groups (N5: 60.5 ± 3.2%; NS: 39.2 ± 4.3%, *P* < 0.05) but not between the N3 (33.3 ± 5.1%) and NS groups (*P* > 0.05). At week 6, muscular strength had recovered to a greater degree in both Neuriitn-treated rats relative to the NS group (N5: 75.0 ± 3.2%, N3: 73.7 ± 3.2%, NS: 45.5 ± 3.2%, *P* < 0.05). At week 8, muscular strength in both Neuritin treatment groups was comparable to that of sham animals. These results provided further evidence that Neuritin treatment accelerates the recovery of injured sciatic nerves.

### The NCV assessment

Nerve conduction velocity (NCV) was reduced in all experimental groups relative to the sham group at all time points after injury (*P* < 0.01), indicating that neural conduction was diminished (Figure 4B). NCV values began to recover by 4 weeks, with significant differences observed between Neuritin and NS groups (N5: 54.21 ± 0.2%, N3: 54.51 ± 4.23%, and NS: 14.74 ± 3.07%; *P* < 0.01). NCV values increased from week 6 to 8 to a greater degree in Neuritin-treated groups (*P* < 0.05); for instance, at week 8, NCV in N5 reached 70% of the original value, which was 2-fold higher than in the NS group (*P* < 0.01).

### Effects of NRN1 treatment on nerve regeneration after sciatic nerve injury

#### Nerve fiber regeneration

**Figure 5:**
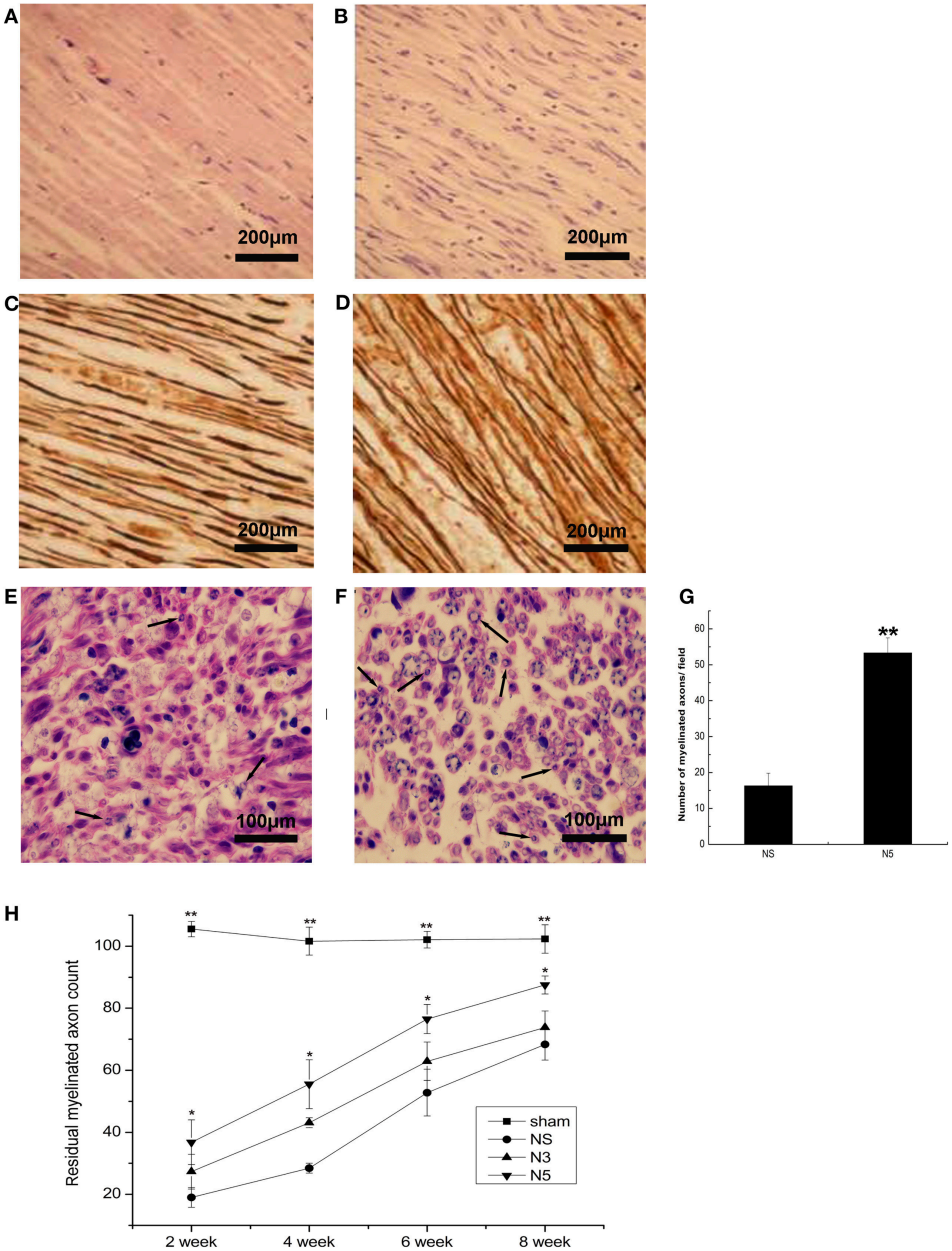
**Regeneration of sciatic nerve fibers after injury. (A,B)** H&E staining of nerve fiber cross sections in NS **(A)** and N5 **(B)** groups. Scale bar = 200 μm. **(C,D)** Sevier-Munger staining in NS **(A)** and N5 **(B)** groups. Scale bar = 200 μm. **(E,F)** Myelin (Solochrome Cyanine R) staining in NS **(A)** and N5 **(B)** groups; arrows indicate newly formed myelin sheaths. Scale bar = 100 μm. **(G)** Quantitative analysis of the number of myelinated axons in each group (*n* = 5). ^**^P < 0.01 vs. NS group. **(H)** Average counts of myelinated nerves in each group at weeks 2, 4, 6, and 8 after injury (*n* = 5). Myelinated fibers were counted at 3 mm distal to the lesion side and the corresponding area in the contralateral side. The average counts of myelinated fibers in each side were calculated as numbers of myelinated fibers/unit area. And then, residual myelinated axon counts were calculated as experimental data/control data × 100%. ^*^P < 0.05, ^**^P < 0.01 vs. NS group.

Hematoxylin and eosin (H&E) staining of sciatic nerve cross sections showed that in the NS group, nerve fibers were loose and curled, with obvious neurotmesis at 2 weeks after injury; there was also negligible nerve regeneration at 4 weeks (Figure 5A). In contrast, nerves had regenerated to a greater degree and showed better organization in the N5 group (Figure 5B). AgNO_3_ staining of nerve fibers showed varying degrees of disarray 2 weeks after injury. At week 4, newly formed nerve fibers were visible in Neuritin-treated animals as compared to those in the NS group, especially the N5 group (Figures 5C,D). At week 6, nerve fibers in the Neuritin groups were well ordered and thicker and more dense than those in the NS group. There was no differences in nerve morphology among groups at week 8.

#### Remyelination

Cross sections of sciatic nerves were stained with Solochrome Cyanine R dye to visualize myelinated nerve fibers. The myelin sheath was disorganized and poorly disordered in all animals 2 weeks after injury. From week 4 to 6, all experimental groups developed myelin sheaths; in the N5 group, these were more strongly stained and better organized than those in the NS group (Figures 5E,F), as quantified in Figure 5G (*P* < 0.01). At week 8, myelin sheaths were thicker in all experimental groups but this change was most significant in the N5 group. The myelin sheath was thin and sparse in the NS group, whereas in the N5 group it was thicker and well organized. The average counts of myelinated fibers were lower in all experimental groups relative to sham animals from week 2 to 8 (*P* < 0.01; Figure 5H). However, by week 4, the average counts of fibers was 2-fold higher in the N5 than in the NS group (N5: 55.5 ± 7.9% and NS: 28.4 ± 1.6%; *P* < 0.05).

#### Axonal regeneration

**Figure 6:**
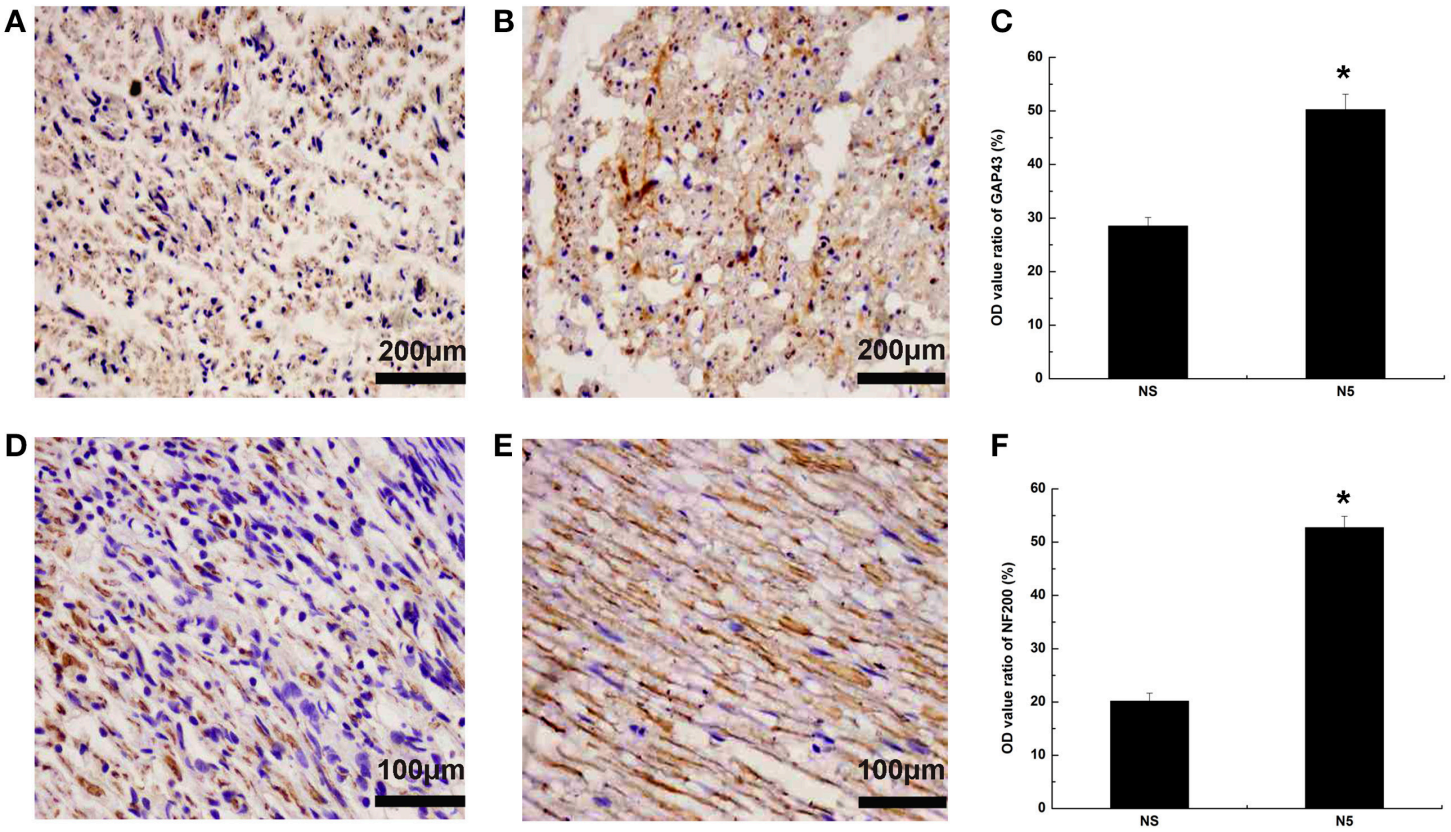
**Axonal regeneration following sciatic nerves injury. (A,B)** GAP43 expression in NS **(A)** and N5 **(B)** groups 4 weeks after injury. Scale bar = 200 μm. **(C)** Quantification of OD ratio for GAP43 expression (*n* = 5). ^*^P < 0.05 vs. NS group. (**D,E**) NF200 expression in NS **(D)** and N5 **(E)** groups 4 weeks after injury. Scale bar = 200 μm. **(F)** Quantification of the OD ratio for NF200 expression (*n* = 5). ^*^P < 0.05 vs. NS group.

To assess the effects of Neuritin on axonal growth, nerve fiber sections were immunolabeled with antibody against the axonal regeneration marker GAP43 4 weeks after injury. GAP43 expression was higher in the N5 group than in the NS group (*P* < 0.05; Figures 6A–C). A similar trend was observed for another axonal regeneration marker NF200 (*P* < 0.05; Figures 6D–F).

## Discussion

In this study we showed that recombinant hNeuritin restored the structure and function of injured sciatic nerve fibers. We used PC12 cells to evaluate the effect of Neuritin on cell morphology. Given the short half-life of neurotrophic factors (Domanskyi et al., 2015), we placed a Neuritin-filled gelatin sponge directly on the site of injury to ensure sustained release, combined with intramuscular injection of Neuritin for 4 weeks to maintain a high circulating level of protein. To exclude inter-individual variability, all indices were calculated by experimental/control data (such as SFI calculation).

To determine whether Neuritin can promote recovery in injured sciatic nerve, we evaluated changes in nerve and muscle function. The SFI was increased by Neuritin at 4 weeks after injury, indicating functional improvement of lower limb muscles and nerves. Gastrocnemius muscle strength and NCV were also improved by Neuritin treatment, with the 5-μg dose having an earlier and more potent effect.

Peripheral nerve function depends on nerve fiber organization (Ivanovic et al., 2012; Gordon et al., 2014). The observed recovery of NCV indicated the restoration of functional nerve fibers; their regular arrangement was confirmed by H&E and silver staining. Moreover, nascent nerve fibers were observed in the Neuritin-treated group. NCV is generated through myelinated nerve fibers; we found that Neuritin treatment restored myelination of injured nerves, thereby accelerating the recovery of NCV.

Since nerve fibers are regenerated from surviving neurons (Xu et al., 2010; Sherpa et al., 2014), we examined changes in the expression of GAP43 and NF200, which are secreted from neurons and act on axons (Liang et al., 2015; Fallini et al., 2016). Neuritin treatment increased the levels of both proteins, possibly by retrograde transport through the lesioned fiber to the cell body where it induced gene expression. A previous study demonstrated that Neuritin is antero- and retrogradely transported along the sciatic nerve *in vivo* (Karamoysoyli et al., 2008). GAP43 is required for growth cone extension and axon guidance, and may induce the expansion of the presynaptic membrane by promoting vesicle fusion or endocytosis at the presynaptic terminal (Di Giovanni et al., 2005a). NF200 is the main cytoskeletal component of axons and is required for stabilization of axonal structure (Black, 2016). During nerve fiber regeneration, GAP43 and NF200 may stimulate the sprouting of axon collaterals and dendrites, thereby increasing the surface area of nerve endings so that new synaptic contacts can be established and contributing to the orderly arrangement of nerve fibers through stabilization of the cytoskeleton.

In conclusion, our results demonstrate that a high concentration of Neuritin in the microenvironment of the injured sciatic nerves slows retrograde degeneration and promotes regeneration. Moreover, we found that Neuritin treatment accelerated the functional recovery of nerve fibers and target tissue. These results highlight the therapeutic potential of Neuritin for the repair of injured nerves.

## Author contributions

JH, LY, and XL conceived and designed the experiments. XL, HW, LS, JZ, and WY performed the experiments. HW and XL analyzed the data. JH, LY, YL, and RC contributed reagents/materials/analysis tools. HW, XL, and JH wrote the paper. All authors read and approved the final manuscript.

## Funding

This work was supported by the Key Project of Xinjiang Province 2014AB048 (JH) and 2008JC11 (JH), the major scientific and technological innovation projects of Hangzhou 20152013A01 (LY). The Program for Zhejiang Leading Team of Science and Technology Innovation (2011R50021); the Foundation of the Most Important Subjects of Hangzhou “Occupational and Environmental Health” (2013–2016).

### Conflict of interest statement

The authors declare that the research was conducted in the absence of any commercial or financial relationships that could be construed as a potential conflict of interest.
