# Corrigendum: Recombinant hNeuritin Promotes Structural and Functional Recovery of Sciatic Nerve Injury in Rats

**DOI:** 10.3389/fnins.2017.00065

**Published:** 2017-02-13

**Authors:** Haiyan Wang, Xinli Li, Liya Shan, Jingling Zhu, Rong Chen, Yuan Li, Wumei Yuan, Lei Yang, Jin Huang

**Affiliations:** ^1^The Key Laboratory of Xinjiang Endemic and Ethnic Diseases and Department of Biochemistry, Shihezi University School of MedicineShihezi, China; ^2^Laboratory Medicine Department of Sixth People's Hospital of ChengduChengdu, China; ^3^Occupational and Environmental Health, Department of Preventive Medicine, School of Medicine, Hangzhou Normal UniversityHangzhou, China; ^4^Department of Otolaryngology Head and Neck Surgery, The Affiliated Hospital of Hangzhou Normal UniversityHangzhou, China

**Keywords:** neuritin, sciatic nerve injury, restoration, walking-track test, growth-associated protein 43, neurofilament 200

**Reason for Corrigendum:**

There was a mistake regarding the affiliation for author Xinli Li. The correct version of author affiliation appears below. The authors apologize for the mistake. This error does not change the scientific conclusions of the article in any way.

**The correct version:**

*Haiyan Wang*^*1*^*, Xinli Li*^*2*^*, Liya Shan*^*1*^*, Jingling Zhu*^*1*^*, Rong Chen*^*3*^*, Yuan Li*^*4*^*, Wumei Yuan*^*1*^*, Lei Yang*^3*^ *and Jin Huang*^1*^

^*2*^*Laboratory Medicine Department of Sixth People's Hospital of Chengdu, Chengdu, China*,

The original article has been updated.

## Conflict of interest statement

The authors declare that the research was conducted in the absence of any commercial or financial relationships that could be construed as a potential conflict of interest.

